# Role of Heat Treatment Atmosphere on the Microstructure and Surface Morphology of DLP-Fabricated High-Entropy Alloy Components

**DOI:** 10.3390/ma18245607

**Published:** 2025-12-13

**Authors:** Jui-Ting Liang, Ting-Hsiang Lin, Vivekanandan Alangadu Kothandan, Shih-Hsun Chen

**Affiliations:** 1 Department of Mechanical and Electro-Mechanical Engineering, National Ilan University, Yilan 260, Taiwan; 2 Department of Mechanical Engineering, National Taiwan University of Science and Technology, Taipei 106, Taiwan; 3 Department of Materials Science and Engineering, National Tsing Hua University, Hsinchu 300, Taiwan; vivekak77@gmail.com; 4 Department of Mechanical Engineering, College of Engineering, National Yang-Ming Chiao Tung University, Hsinchu 300, Taiwan

**Keywords:** high-entropy alloys (HEAs), AlCrFeNiSi, digital light processing (DLP), heat treatment atmosphere, gas atomization

## Abstract

AlCrFeNiSi high-entropy alloy (HEA) components were fabricated using digital light processing (DLP) 3D printing, followed by debinding under oxygen-rich and oxygen-deficient atmospheres and sintering at various temperatures. The influence of atmosphere on microstructural evolution, elemental redistribution, and mechanical consolidation was systematically investigated. Oxygen-rich debinding induced oxidation-driven gas formation and surface cracking, whereas oxygen-deficient debinding preserved residual carbon that reduced porosity and enabled earlier densification. The layered microstructure progressively vanished with temperature, and full consolidation was achieved at 1100 °C in oxygen-rich and 1050 °C in oxygen-deficient environments. Correspondingly, both processing conditions yielded similar maximum compressive strengths (~5 MPa), although the oxygen-deficient condition attained this strength at a lower temperature. These findings demonstrate that controlling oxygen exposure during debinding provides an effective pathway to reduce the sintering temperature while maintaining the mechanical performance of DLP-printed AlCrFeNiSi HEA components.

## 1. Introduction

The evolution of human technology has led to four major industrial revolutions, with Industry 4.0 emphasizing smart manufacturing to meet the growing demand for customized, multifunctional products. Among the ten key technological domains proposed for Industry 4.0, advanced materials and three-dimensional (3D) printing technologies have emerged as pivotal in the mechanical and materials fields due to their ability to deliver high precision and versatile performance.

Traditional alloys, typically dominated by a single principal element (>50 at.%), often fail to simultaneously meet critical requirements such as high-temperature stability and corrosion resistance, restricting their practical applications. To overcome these constrains, high-entropy alloys (HEAs) were independently introduced in 2004 by Professor Brian Cantor (University of Oxford) and Professor Jien-Wei Yeh (National Tsing Hua University) [1,2]. HEAs are generally defined as alloys containing at least five principal elements, each with an atomic fraction of 5–35 at.% [3,4]. The absence of a dominant element promotes strong synergistic effects among the constituent elements, enabling exceptional mechanical strength, thermal stability, and corrosion resistance [3,5,6].

Early research on HEA focused on identifying element combinations capable of forming stable solid-solution phases. The CoCrFeMnNi alloy (commonly known as the Cantor alloy) is the most extensively studied face-centered cubic (FCC) system, noted for its excellent cryogenic ductility and fracture toughness [1,7], although it exhibits limited wear and corrosion resistance [8]. Subsequent efforts introduced compositional tuning to enhance specific properties [8]. For instance, manganese additions can alter lattice structure while simultaneously promoting oxidation and oxide spallation [9,10,11], whereas aluminum promotes body-centered cubic (BCC) phase formation and lattice distortion, thereby improving hardness and oxidation resistance [12,13]. To further reduce cost and improve processability, recent studies have explored cobalt-free systems [14,15,16]. Moreover, incorporating non-metallic elements such as silicon has been shown to enhance hardness, compressive strength, and wear resistance [17,18,19,20,21], motivating the present focus on the AlCrFeNiSi system.

Conventional bulk-fabrication of HEAs often suffers from sluggish diffusion and compositional segregation during cooling [22]. To mitigate these issues, research has increasingly shifted toward powder-based processing, where fine powders shorten diffusion paths and promote densification [23]. Gas atomization enables the production of homogeneous, flowable powders with minimal contamination and suppressed segregation [24], which are well suited for advanced techniques such as additive manufacturing [25,26] and thermal spraying [27]. Fine powders not only improve heat transfer while facilitating the production of high-value, small-scale functional components—such as catalysts and porous structures [28,29,30]—thereby highlighting the potential of additive manufacturing for HEA applications.

Additive manufacturing offers inherent advantages, including customization, high dimensional accuracy, and the ability to produce complex geometries, effectively mitigating the coarse grains or compositional segregation commonly encountered in conventional processes [23] and shortening the product-development cycle. Nevertheless, mainstream metallic additive-manufacturing techniques, such as fused-deposition modeling [31] and laser sintering [32], often generate significant residual thermal stresses during fabrication, which can degrade mechanical properties and increase processing complexity. Therefore, the development of novel manufacturing routes that minimize thermal-stress-induced defects is imperative. Digital light processing (DLP), a photopolymerization-based technique [33], fabricates components without the high thermal loads typical of powder-bed fusion, thereby avoiding residual stress while enabling high build rates. DLP has demonstrated promising results and applied in the ceramic powder processing [34], and its integration with HEA powders offers strong potential for the precision fabrication of high-value miniature parts.

In this context, the present study employs gas atomization to produce compositionally homogeneous AlCrFeNiSi powders, which are subsequently blended with a photocurable resin to prepare a light-curable composite slurry for DLP-based 3D printing of HEA components. The printed green bodies are debinded and sintered under both oxygen-rich and oxygen-deficient atmospheres. A systematic investigation is conducted to elucidate the relationships among processing parameters, powder–resin bonding behavior, microstructural evolution, elemental distribution, and mechanical properties. Furthermore, cross-sectional microstructures of samples subjected to various heat treatment conditions are examined to assess the effects of atmosphere and temperature on internal densification and pore distribution. By optimizing heat treatment parameters, this work aims not only to achieve higher mechanical strength but also to provide fundamental insights into the microstructural evolution mechanisms during sintering, thereby enhancing the industrial applicability of high-entropy alloys.

## 2. Experimental Procedures

The as-atomized powders with homogeneous composition were first produced via gas atomization and subsequently mixed with a photocurable resin through mechanical stirring to prepare a printable composite slurry. The slurry was then used to fabricate specimens by digital light processing (DLP)-based 3D printing (FREENTIT Co., Ltd., Taipei, Taiwan). After printing, the green bodies underwent debinding and sintering under different heat treatment conditions. Following thermal processing, the specimens were characterized through microstructural observations, surface morphology analysis, and compressive mechanical testing. In addition, elemental diffusion behavior across powder interfaces was examined to elucidate the influence of heat treatment on microstructural evolution and mechanical performance.

### 2.1. Raw AlCrFeNiSi High-Entropy Alloy Powder

The AlCrFeNiSi high-entropy alloy powders (Chung-Yo Materials Co., Ltd., Kaohsiung, Taiwan) used in this study were produced by gas atomization and subsequently sieved to obtain a particle size range of 10–60 μm. The overall morphology of the powders was first examined at low magnification using a scanning electron microscope (SEM) (JEOL, Tokyo, Japan), while their elemental composition and distribution were analyzed by energy-dispersive X-ray spectroscopy (EDS) (Oxford, Oxfordshire, England). The potential for solid-solution formation and the valence electron concentration (VEC) of the alloy system were then calculated using established empirical formulas to assess its crystallographic structure tendency. Finally, the melting point of the powders was determined by simultaneous thermal analysis (STA), which provided the basis for selecting subsequent heat treatment parameters.

### 2.2. Preparation of Photocurable Resin Slurry

The photocurable resin used in this study was composed of an oligomer, a monomer, and a photoinitiator, which were thoroughly mixed and then blended with the AlCrFeNiSi high-entropy alloy powders to prepare the printable slurry. The oligomer was an acrylate-based material (DOUBLEMER 7201M) (Double Bond Chemical Ind. Co., Ltd., New Taipei City, Taiwan), an aromatic urethane diacrylate diluted with 20 wt.% isobornyl acrylate (IBOA) (boiling point ≈ 450 °C), which cured under ultraviolet (UV) or electron-beam irradiation and exhibited good flexibility but required preheating to 50 °C before use. The monomer was a trifunctional acrylate (DM TMPTA) (Double Bond Chemical Ind. Co., Ltd., New Taipei City, Taiwan) with a boiling point of ≈280.9 °C, which polymerized rapidly under UV irradiation, producing a highly cross-linked network with fast curing, high hardness, strong gloss, and excellent solvent and chemical resistance. The photoinitiator was diphenyl (2,4,6-trimethylbenzoyl) phosphine oxide (TPO), a white to light-yellow powder with an absorption range of 379–387 nm; upon UV exposure, it decomposed to generate free radicals and initiate polymerization, exhibiting high curing activity and low toxicity while improving the wear resistance, corrosion resistance, and adhesion of the cured resin. All resin materials were supplied by Double Bond Chemical Ind. Co., Ltd. (New Taipei City, Taiwan).

To ensure homogeneous mixing, the monomer, oligomer, and photoinitiator were first blended using a mechanical stirrer to obtain a uniform photocurable resin. The resin was subsequently mixed with the AlCrFeNiSi high-entropy alloy powders using a planetary centrifugal mixer (ARE-310, Thinky Corp., Laguna Hills, CA, USA). The slurry composition is summarized in Table 1, where the oligomer accounted for approximately 60.5 vol.% of the resin to enhance the overall strength, while 9.2 vol.% of the photoinitiator was added to improve curing efficiency. The corresponding operating parameters of the mixing equipment are listed in Table 2.

**Figure 1:**
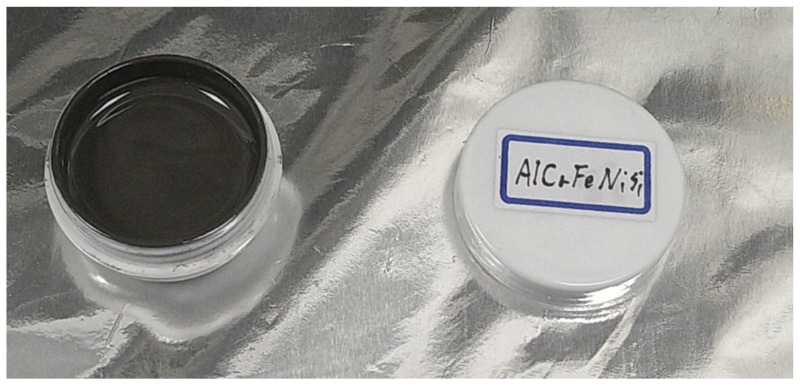
Mixing slurry with 60 vol.% HEA powder.

During processing, the resin underwent a pre-stirring treatment for approximately two days at a controlled temperature of 50 °C to ensure uniformity. The subsequent planetary centrifugal mixing was performed at a rotation speed of 2000 rpm for 4 min, yielding the final photocurable slurry. The morphology of the prepared slurry is shown in Figure 1.

### 2.3. Parameters of DLP

Digital light processing (DLP) is a widely used 3D printing technique that employs a light source to irradiate photocurable materials, inducing photochemical reactions to solidify the resin layer by layer until a complete three-dimensional object is constructed. In this study, a customized DLP printer (resolution: 25 μm) provided by Freentity Co. (Taipei, Taiwan) was employed to fabricate specimens using the photocurable slurry containing AlCrFeNiSi high-entropy alloy powders.

**Figure 2:**
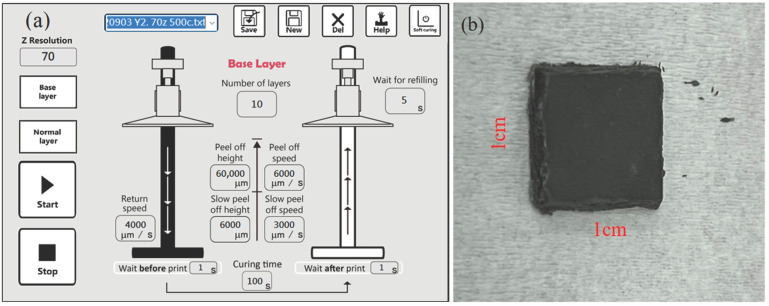
The (**a**) detailed printing configuration and (**b**) the appearance of green body.

During printing, the designed 3D model was converted into a machine-readable format, with an in-plane dimension of 1 cm × 1 cm, a layer thickness of 70 μm, and a total of 20 layers. Printing parameters were progressively optimized through multiple trials, as summarized in Table 3. The printer utilized a 405 nm ultraviolet light source, and the light intensity was measured as 19.57 mW/cm^2^ using an optical power density meter. To ensure sufficient interlayer curing, the exposure time was set to 100 s, resulting in the curing depth of 100 μm. After printing, the specimens were cleaned in ethanol by ultrasonic agitation to remove uncured resin. In addition, to prevent delamination caused by excessive platform lifting speed, the peel-off speed was maintained at 100 μm/s. The detailed printing configuration and green body are illustrated in Figure 2.

### 2.4. Heat Treatment

The printed specimens were subjected to debinding and sintering in a tubular high-temperature furnace. Debinding was a critical and delicate process, as inappropriate heating cycles could lead to defects such as cracking, blistering, or collapse due to non-uniform thermal stresses. Therefore, thermal analysis was first performed to determine suitable parameters.

**Figure 3:**
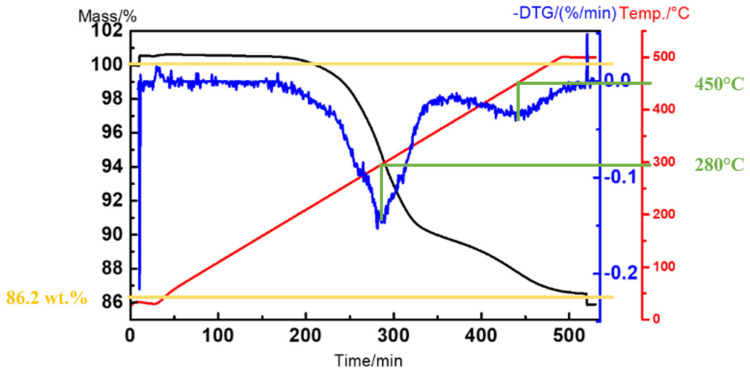
Thermogravimetric analysis of slurry.

As shown in Figure 3, the thermogravimetric analysis (TGA) revealed two distinct stages of weight loss during heating, with a total mass reduction of 13.8 wt.% and a final residual weight of approximately 86.2 wt.% (≈60 vol.%), which was consistent with the estimated solid loading of the slurry. The derivative thermogravimetric (DTG) curve further indicated two major decomposition peaks at around 280 °C and 440 °C, corresponding to the degradation of the oligomer (7201M) and the monomer (TMPTA), respectively.

**Figure 4:**
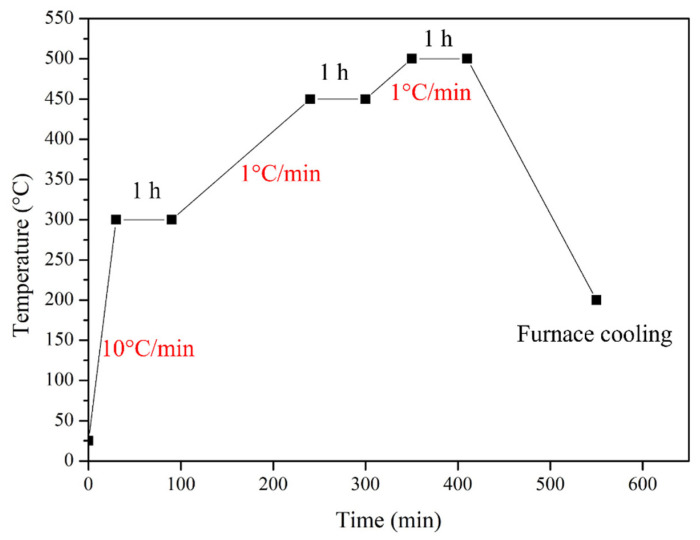
Debinding heating curve.

Based on these results, the debinding schedule was set to include isothermal holds at 300 °C and 450 °C for 1 h each to ensure sufficient decomposition of the organic components, followed by heating to 500 °C with an additional dwell to complete the debinding process. The samples were subsequently cooled to room temperature inside the furnace. The detailed debinding temperature profile is presented in Figure 4.

The heat treatment parameters adopted in this study, including temperature, dwell time, and atmospheric conditions, are summarized in Table 4. Experiments were conducted under two distinct atmospheres: (i) an oxygen-deficient environment, maintained by continuous evacuation using a mechanical pump to reduce oxygen content, and (ii) an oxygen-rich environment, carried out directly in ambient air. All heat treatments were performed in a tubular high-temperature furnace with precise temperature and time control.

According to the principles of solid-state sintering, the sintering temperature is typically selected within the range of approximately 2/3 to 4/5 of the material’s melting point. The tubular furnace employed in this study was capable of reaching 1200 °C, which was also close to the measured melting point of the AlCrFeNiSi high-entropy alloy powders. To investigate the microstructural evolution at different thermal conditions, sintering dwell temperatures were set to 800 °C, 1000 °C, 1050 °C, and 1100 °C, each maintained for 24 h. Under oxygen-deficient conditions, vacuum pumping was sustained throughout the process to minimize oxidation.

## 3. Results and Discussion

### 3.1. Effect of Debinding Atmosphere on the Microstructure and Elemental Distribution of High-Entropy Alloys

This section focuses on the debinding atmosphere as the primary variable to investigate the influence of oxygen-rich and oxygen-deficient environments on the microstructural evolution of DLP-printed green bodies. Scanning electron microscopy (SEM) combined with energy-dispersive X-ray spectroscopy (EDS) elemental mapping is employed to examine the microstructural characteristics and elemental distribution of the samples after debinding. This analysis provides insight into resin removal efficiency and initial interparticle diffusion behavior under different atmospheric conditions. The most critical finding is the role of oxygen in forming effective removal of carbonaceous binders on the AlCrFeNiSi HEA powders, which significantly dictates subsequent sintering behavior.

**Figure 5:**
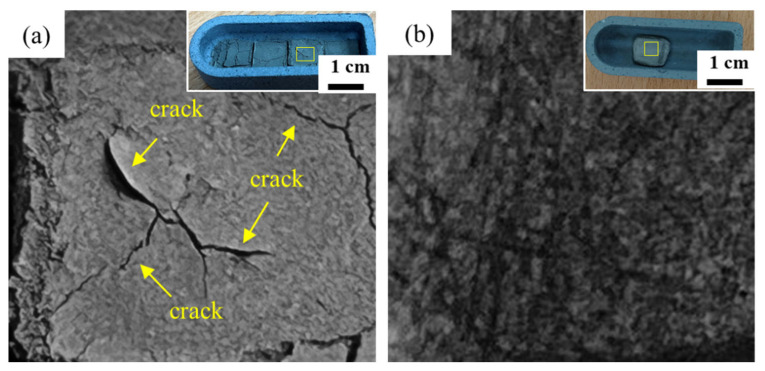
Macroscopic morphology of 3D-printed samples after debinding under different atmospheres: (**a**) oxygen-rich and (**b**) oxygen-deficient conditions. The inserted image represents the appearance of the original sample, and the selected area indicates the magnified region.

As shown in Figure 5a, under an oxygen-rich debinding environment, sufficient oxygen supply promotes oxidation reactions between the resin and metal powders. The visible crack formed in the oxygen-rich environment is due to the rapid internal pressure buildup of CO/CO_2_ gas escaping through a weakened high-porosity structure. In contrast, when debinding in oxygen-deficient conditions, the inadequate oxygen availability prevents complete oxidation of the carbon within the resin, leaving carbon residues in the original resin regions. As a result, gas evolution is significantly diminished, and no cracks are observed on the sample surface shown in Figure 5b. These findings suggest that the observed crack formation under oxygen-rich conditions is caused by rapid gas release, which implies that adopting a slower, controlled heating rate during debinding is necessary to ensure the structural integrity of the green body.

**Figure 6:**
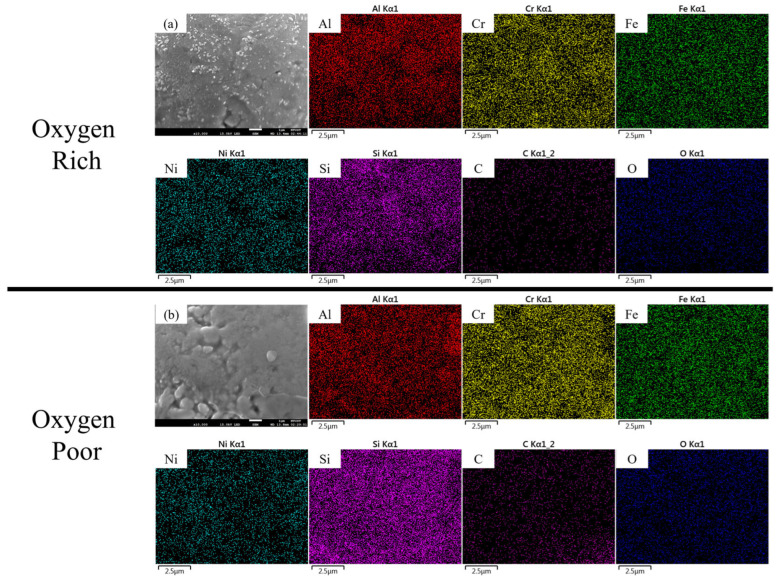

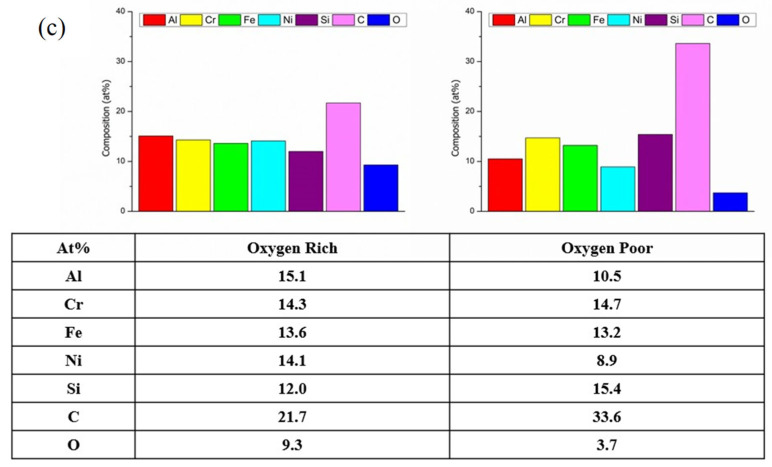
Microstructure and elemental distribution of 3D-printed samples after debinding under different atmospheres: (**a**) oxygen-rich, (**b**) oxygen-deficient, and (**c**) corresponding elemental composition variations and elemental composition quantitative data.

After debinding, the specimens are mounted, ground, and polished for detailed SEM and EDS analyses to evaluate elemental composition and distribution. As shown in Figure 6a, samples debinded in the oxygen-rich atmosphere exhibit a higher oxygen content and lower residual carbon, while the AlCrFeNiSi HEA powders remain uniformly distributed. Conversely, in Figure 6b, the oxygen-deficient atmosphere results in incomplete oxidation and higher carbon retention in the former resin regions; nevertheless, the high-entropy alloy particles display a homogeneous dispersion throughout the matrix.

The chemical analysis shown in Figure 6c and the corresponding quantitative data in Table 1 confirm the profound influence of the debinding atmosphere on the AlCrFeNiSi HEA powders. As clearly indicated by the bar chart, the oxygen-rich environment resulted in the higher mean oxygen content (Oxygen 9.3 at.%) across the sample cross-section, which is almost three times more than that of the oxygen-deficient condition (Oxygen 3.7 at.%). Thus, the oxygen availability significantly lower-residual carbon content (21.7 at.%) in the oxygen-rich samples, compared to the 33.6 at.% left in the oxygen-deficient atmosphere.

### 3.2. Effect of Heat Treatment Temperature on the Sintering Behavior

#### 3.2.1. Oxygen-Rich Atmosphere

**Figure 7:**
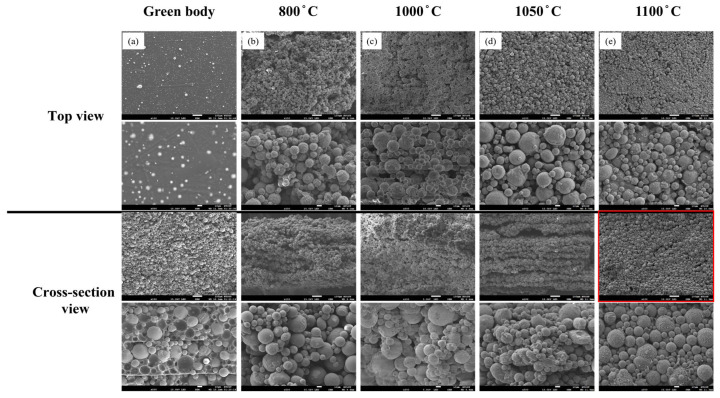
SEM micrographs of the top and cross-sectional surfaces of 3D-printed samples debinded in an oxygen-rich atmosphere and sintered at different temperatures for 24 h: (**a**) green body, (**b**) 800 °C, (**c**) 1000 °C, (**d**) 1050 °C, and (**e**) 1100 °C. (The red frame highlight an obvious morphology change.).

The influence of heat treatment temperature on the sintering behavior of the specimens was examined in the range of 800–1100 °C, under identical holding times of 24 h. All samples were debinded under oxygen-rich conditions prior to heat treatment to ensure a consistent pre-sintering state. The evolution of surface morphology and interparticle bonding was characterized using SEM observations on both top and side surfaces to evaluate the degree of densification (Figure 7).

At 600 °C, the samples exhibited disintegration and loss of structural integrity, indicating that this temperature was insufficient for initiating effective solid-state sintering. Therefore, subsequent analyses focused on the temperature range between 800 °C and 1100 °C. As the temperature increases, the interparticle distance gradually decreases and the particle boundaries become less distinct, signifying enhanced densification. At 1100 °C, the initial layered structure disappeared and transformed into a compact structure. The enhanced bonding between adjacent particles at elevated temperatures can be attributed to thermally activated solid-state diffusion, which promotes mass transport across particle interfaces.

**Figure 8:**
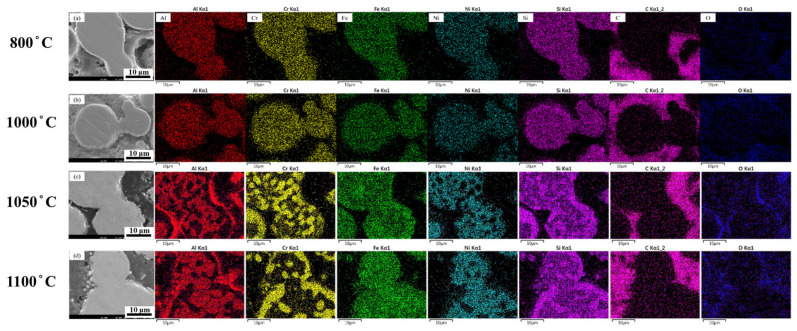
Microstructure and elemental distribution of 3D-printed samples debinded in an oxygen-rich atmosphere and sintered at different temperatures for 24 h: (**a**) 800 °C, (**b**) 1000 °C, (**c**) 1050 °C, and (**d**) 1100 °C.

Further analysis is carried out using SEM combined with EDS elemental mapping to investigate the microstructural evolution and elemental distribution of specimens subjected to different heat treatment temperatures. This analysis provides insights into the interparticle bonding mechanism and elemental diffusion behavior. As shown in Figure 8, all specimens are debinded under oxygen-rich conditions prior to heat treatment, with a fixed holding time of 24 h.

When the sintering temperature is below 1000 °C, the thermal energy is insufficient to drive substantial elemental diffusion. Consequently, both the microstructure and elemental distribution remain relatively uniform, and the interfaces between adjacent particles are still clearly distinguishable. At 1050 °C, the increased thermal energy provides a stronger diffusion driving force, promoting interparticle elemental exchange. As a result, the particle boundaries gradually disappear, indicating improved interfacial bonding. In addition, EDS mapping of aluminum and oxygen revealed that these elements combined preferentially at the outer regions of the powder particles, forming localized oxides that accumulate on the particle surfaces. Thus, the formation of a dense oxide layer acts as a protective barrier against further oxidation, thereby improving material stability at elevated temperatures.

**Figure 9:**
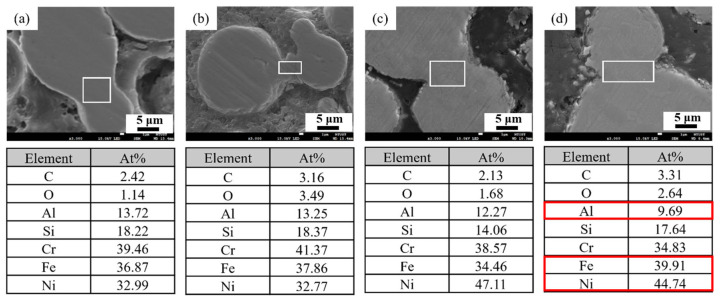
EDS compositional analysis of 3D-printed samples debinded in an oxygen-rich atmosphere and sintered at different temperatures for 24 h: (**a**) 800 °C, (**b**) 1000 °C, (**c**) 1050 °C, and (**d**) 1100 °C. (The white frame in each figure presents the area of obtaining composition data.).

The influence of sintering temperature on the elemental distribution at the powder–particle interfaces is further examined, and the results are presented in Figure 9. As the temperature increased to 1100 °C, a noticeable decrease in the aluminum concentration was observed, indicating pronounced elemental interdiffusion between adjacent particles. Meanwhile, elements with relatively lower diffusion rates, such as nickel, showed increased interfacial enrichment at 1050 °C. This indicates that the thermal energy at this temperature is sufficient to promote the diffusion of internal elements toward the particle boundaries. These observations confirm that interparticle elemental exchange becomes more pronounced at elevated temperatures, enhancing interfacial bonding and overall consolidation. This, in turn, contributes to improved densification and mechanical performance of the material.

#### 3.2.2. Oxygen-Deficient Atmosphere

**Figure 10:**
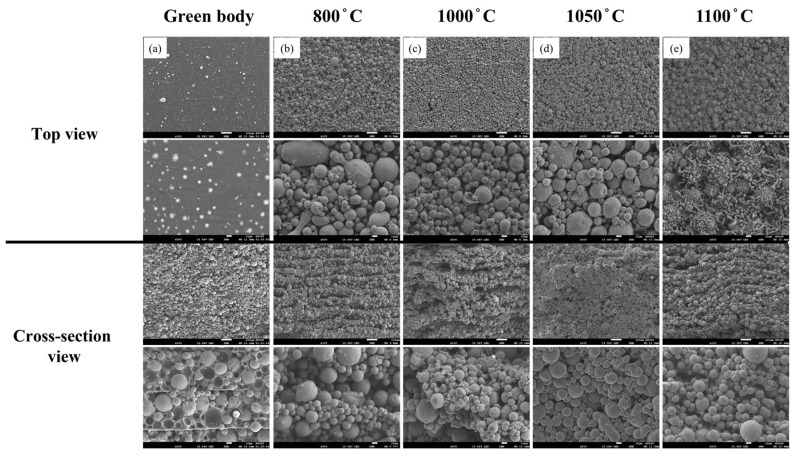
SEM micrographs of the top and cross-sectional surfaces of 3D-printed samples debinded under oxygen-deficient conditions and sintered at different temperatures for 24 h: (**a**) green body, (**b**) 800 °C, (**c**) 1000 °C, (**d**) 1050 °C, and (**e**) 1100 °C.

The microstructural evolution of the samples debinded under oxygen-deficient conditions and subsequently sintered at different temperatures for 24 h is examined using SEM (Figure 10). As the sintering temperature increased, the original layered structure gradually became indistinct, indicating that the degree of interparticle bonding strengthens with increasing temperature. At 1050 °C (Figure 10d), the layered morphology nearly disappears, and the specimen significantly improved compactness, accompanied by a marked reduction in the interparticle spacing. These observations suggest that both diffusion and bonding effects become more pronounced during the sintering process at elevated temperatures.

When the temperature further increases to 1100 °C (Figure 10e), stripe-like precipitates appeared on the particle surfaces. This phenomenon is likely attributed to the absence of an oxide protective layer on the fine powder particles under oxygen-deficient conditions, which results in localized surface softening or melting of the smaller particles and their subsequent adhesion to the surfaces of larger particles, forming elongated precipitates.

**Figure 11:**
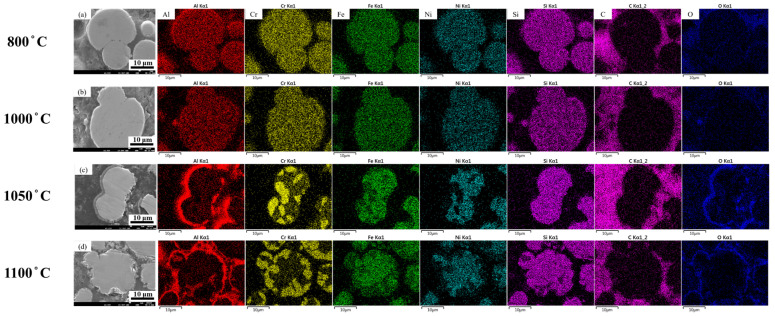
Microstructure and elemental distribution of 3D-printed samples debinded in an oxygen-deficient atmosphere and sintered at different temperatures for 24 h: (**a**) 800 °C, (**b**) 1000 °C, (**c**) 1050 °C, and (**d**) 1100 °C.

Similarly to the analysis performed under oxygen-rich conditions, SEM observation combined with EDS elemental mapping were conducted to examine the microstructure and elemental distribution of the samples debinded under oxygen-deficient conditions and sintered at various temperatures for 24 h (Figure 11). When the holding temperature was below 1000 °C, the material lacked sufficient thermal energy to drive noticeable elemental diffusion; thus, the microstructure and elemental distribution remained uniform, and the particle interfaces were still clearly distinguishable. As the temperature rises to 1050 °C, the increased thermal energy provided a stronger diffusion driving force, promoting elemental exchange between adjacent particles. The particle boundaries gradually disappeared, indicating enhanced interfacial bonding and cohesion. When the temperature further increased to 1100 °C, elemental diffusion became more pronounced, and certain regions exhibited localized enrichment, transitioning from a uniform to a segregated elemental distribution. These results indicate that, under oxygen-deficient conditions, diffusion becomes increasingly interface-dominated at higher temperatures.

**Figure 12:**
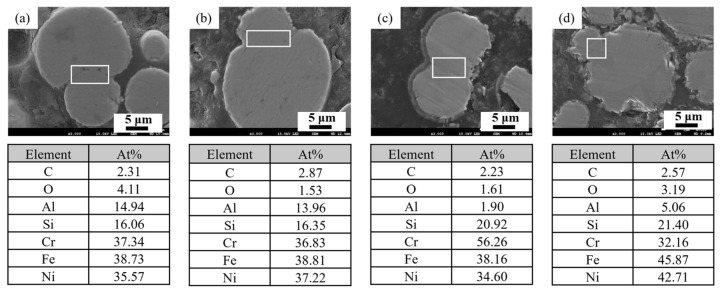
EDS compositional analysis of 3D-printed samples debinded in an oxygen-deficient atmosphere and sintered at different temperatures for 24 h: (**a**) 800 °C, (**b**) 1000 °C, (**c**) 1050 °C, and (**d**) 1100 °C. (The white frame in each figure presents the area of obtaining composition data.)

To examine temperature-dependent elemental redistribution, Figure 12 displays the EDS maps of oxygen-deficient samples sintered at 800, 1000, 1050, and 1100 °C. Unlike the samples debinded in oxygen-rich environments, the aluminum content decreases noticeably at 1050 °C, indicating substantial interparticle elemental exchange. Moreover, for elements with relatively low diffusion rates such as nickel, their interfacial concentration increased significantly at 1100 °C, suggesting that high thermal energy facilitates internal elemental diffusion towards the particle boundaries.

### 3.3. Correlation Between Heat Treatment Parameters and the Mechanical Properties of High-Entropy Alloys

**Figure 13:**
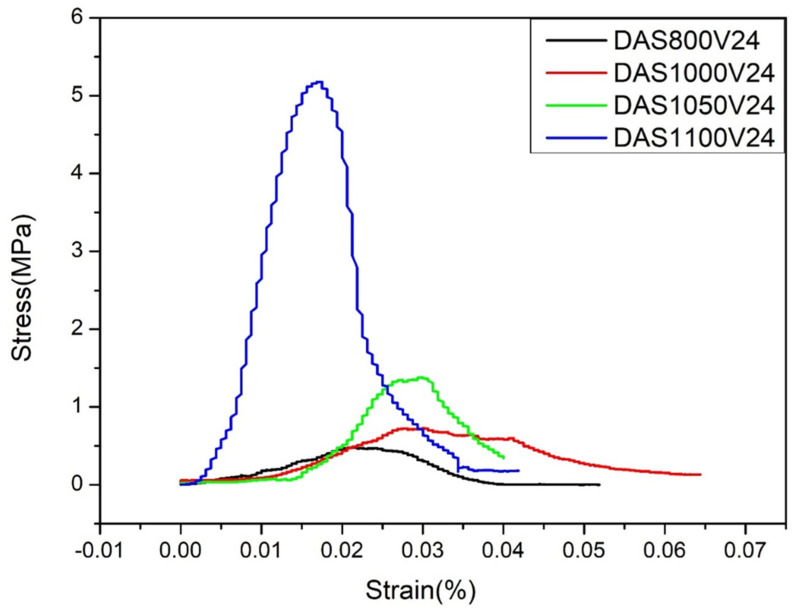
Mechanical property analysis of 3D-printed samples debinded in an oxygen-rich atmosphere and sintered at different temperatures for 24 h.

In accordance with the preceding microstructural and elemental analyses, this section evaluates the influence of sintering temperature and debinding atmosphere on the mechanical behavior of the 3D-printed AlCrFeNiSi specimens. The compressive strength of samples subjected to different heat treatment conditions was measured using an MTS 42.503 universal testing machine (MTS Systems, Prairie, MN, USA). As shown in Figure 13, the compressive strength of the samples debinded in an oxygen-rich atmosphere increased progressively with sintering temperature, reaching 5.17 MPa at 1100 °C. This mechanical response aligns with the corresponding SEM cross-sectional observation, which shows that complete elimination of the layered structure occurs only at 1100 °C, indicating full structural consolidation at this temperature.

**Figure 14:**
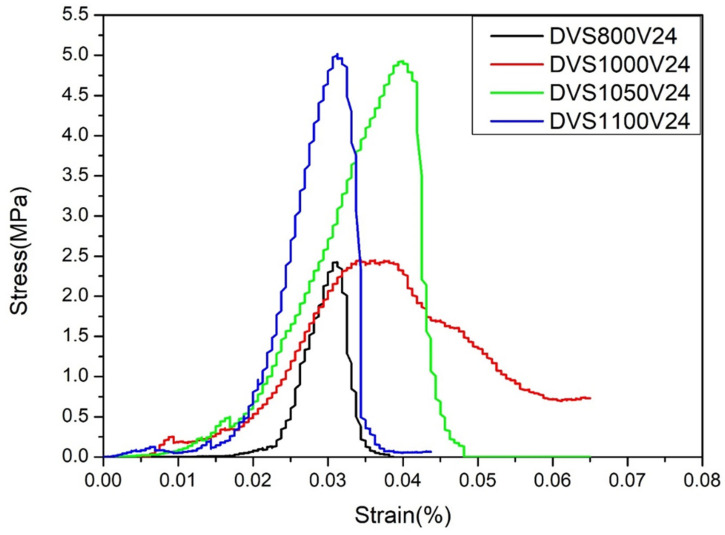
Mechanical property analysis of 3D-printed samples debinded in an oxygen-deficient atmosphere and sintered at different temperatures for 24 h.

Under oxygen-deficient conditions (Figure 14), the compressive strength increased noticeably even at lower temperatures. The early development of high strength (≈800 °C) corresponds with the microstructural evolution of these specimens, which show improved particle bonding and reduced porosity at lower temperatures. This behavior is attributed to residual carbon retained during debinding, which decreases pore volume and enhances the initial load-bearing capability. A maximum compressive strength of 4.93 MPa was reached at 1050 °C, and the increase at 1100 °C was minimal (5.1 MPa), indicating that the specimens approached densification saturation, where further heating yields limited mechanical improvement. At 1050 °C, the layered structure disappeared, and material density increased substantially, further contributing to the strength improvement. In contrast, samples debinded in oxygen-rich conditions required a higher sintering temperature (1100 °C) to attain comparable compressive strength, demonstrating that oxidation-related interfacial resistance delays effective particle bonding and densification. These results suggest that eliminating surface oxidation promotes interparticle diffusion and bonding, while oxygen-rich atmospheres generate oxide layers that impede densification and delay structural consolidation.

## 4. Conclusions

AlCrFeNiSi high-entropy alloy powders produced by gas atomization are mixed with a photocurable resin and fabricated via DLP 3D printing, followed by debinding under oxygen-rich and oxygen-deficient atmospheres and sintering at various temperatures.

The key findings are summarized below:

(1) After debinding, the elemental distribution of the powders remained uniform. In oxygen-rich environments, oxidation between the resin and oxygen produces gases that induce surface cracking, requiring a slower heating rate to avoid defects. The layered structure becomes indistinct with increasing temperature and disappears at 1100 °C for oxygen-rich and at 1050 °C for oxygen-deficient samples. In oxygen-deficient atmosphere, stripe-like precipitates form at 1100 °C due to local softening or melting of fine particles in the absence of oxide protection.
(2) Elemental analyses revealed that aluminum formed surface oxides at 1050 °C under oxygen-rich conditions, contributing to interparticle bonding, whereas under oxygen-deficient conditions, residual carbon facilitated diffusion and bonding at lower temperatures. Oxygen-deficient debinding combined with moderate sintering (~1050 °C) promotes efficient diffusion and interfacial bonding comparable to high-temperature (1100 °C) oxygen-rich sintering.
(3) Compressive strength increased with sintering temperature for both atmospheres. Under oxygen-deficient conditions, strength increased from 2.4 MPa at 800 °C to 5.0 MPa at 1100 °C; for oxygen-rich samples, it increased from 0.5 MPa to 5.1 MPa. Comparable strength was achieved at 1050 °C under oxygen-deficient conditions and 1100 °C under oxygen-rich conditions, demonstrating that earlier diffusion-driven consolidation improves mechanical performance.

Overall, this work provides one of the first systematic comparisons of oxygen-rich and oxygen-deficient debinding pathways in DLP-printed HEA powders, establishing clear correlations among sintering atmosphere, interfacial diffusion, and mechanical consolidation.

## Figures and Tables

**Table 1:** The composition of photocurable slurry.

Instrument	Type	Name	Composition
Mechanical stirrer	Oligomer	7201M	60.5 vol.%
	Monomer	DM TMTPA	30.3 vol.%
	Photo initiator	TPO	9.2 vol.%
Planetary centrifugal mixer	Mixing resin	7201M/TMTPA/TPO	40 vol.%
	HEAs powder	AlCrFeNiSi	60 vol.%

**Table 2:** Parameters of slurry mixing.

Mechanical Stirrer	
Temperature (°C)	50
Time (day)	2
Planetary centrifugal mixer	
Speed (rpm)	2000
Time (min)	4

**Table 3:** DLP Process Parameters.

Printing Parameters	
Lighting Power Density (mW/cm^2^)	19.57
Wavelength of UV (nm)	405
Curing time (s)	100
Resolution (μm)	25
Curing depth (μm)	100
Post treatment	
Solvent	Ethanol
Time (s)	30
Temperature (°C)	Room temperature

**Table 4:** Parameter of heat treatment.

	Debinding	Sintering
Temperature (°C)	300, 450, 500	800, 1000, 1050, 1100
Duration (hour)	1	24
Atmosphere	oxygen-deficient, oxygen-rich	oxygen-deficient
Cooling method	Furnace cooling	

## Data Availability

The original contributions presented in this study are included in the article. Further inquiries can be directed to the corresponding author.
